# Structure and Molecular Mechanism of ER Stress Signaling by the Unfolded Protein Response Signal Activator IRE1

**DOI:** 10.3389/fmolb.2019.00011

**Published:** 2019-03-12

**Authors:** Christopher J. Adams, Megan C. Kopp, Natacha Larburu, Piotr R. Nowak, Maruf M. U. Ali

**Affiliations:** Department of Life Sciences, Imperial College London, London, United Kingdom

**Keywords:** unfolded protein response (UPR), IRE1 inositol-requiring enzyme 1, ER stress, crystal structures, Hsp70, BiP

## Abstract

The endoplasmic reticulum (ER) is an important site for protein folding and maturation in eukaryotes. The cellular requirement to synthesize proteins within the ER is matched by its folding capacity. However, the physiological demands or aberrations in folding may result in an imbalance which can lead to the accumulation of misfolded protein, also known as “ER stress.” The unfolded protein response (UPR) is a cell-signaling system that readjusts ER folding capacity to restore protein homeostasis. The key UPR signal activator, IRE1, responds to stress by propagating the UPR signal from the ER to the cytosol. Here, we discuss the structural and molecular basis of IRE1 stress signaling, with particular focus on novel mechanistic advances. We draw a comparison between the recently proposed allosteric model for UPR induction and the role of Hsp70 during polypeptide import to the mitochondrial matrix.

## Introduction

The endoplasmic reticulum is a major site for protein folding and maturation within the eukaryotic cell. Proteins that reside in the ER, along with proteins destined for the Golgi, plasma membrane, and extracellular space are synthesized in ribosomes that are attached to the ER membrane. The newly translated polypeptide contains an N-terminal signal sequence that is recognized by signal recognition particle (SRP), which enables its insertion into the ER via the transolocon complex. Once inside the ER, the signal sequence is cleaved by signal peptidase and the translocated polypeptide undergoes post-translational modification and chaperone-assisted folding to help it to form its correct three-dimensional shape. There are various ER resident enzymes and chaperones that increase the efficiency of protein folding of the nascent polypeptide. One of the most abundant proteins within the ER is the Hsp70-type chaperone, BiP (binding-immunoglobulin protein aka GRP-78). BiP binds to nascent polypeptide chains to prevent their aggregation initially; and subsequently, facilitates their folding in order for the polypeptide to achieve its native conformation. Post-translational modifications are also critical for correct protein folding and one such modification is disulphide bond formation. This bond is important for maintaining tertiary and quaternary protein structure and is catalyzed by protein disulphide isomerase (PDI). Another essential modification is the attachment of N-linked oligosaccharides to the nascent chain, which occurs upon entry into the ER. Once in the ER, there are a number of glycosylating enzymes that either trim or add to the core N-linked oligosaccharide depending on the progress of protein folding. These alterations to the glycan chain help to monitor the folding status of the nascent polypeptide and act as an important quality control measure (Wang and Kaufman, 2016; Hetz and Papa, 2017).

A failure in the polypeptide chain to adopt its native conformation may lead to activation of degradation pathways, including ER-associated degradation (ERAD) (Hampton, 2002). In this process, misfolded protein is retro-translocated across the ER membrane into the cytosol, where it is ubiquitylated and targeted for degradation via the 26S proteasome. However, if the polypeptide adopts its correct shape, it can then transit to the Golgi and advance further through the secretory pathway.

The environment within the ER is more oxidizing than that of the cytosol. This is conducive to the formation of disulphide bonds, which occurs predominantly within the ER. Furthermore, a high concentration of calcium helps to buffer protein folding especially since many ER chaperones require calcium as a co-factor to operate effectively.

Protein folding requirements within the ER vary depending on cell type. For specialized secretory cells, such as plasma cells, insulin-producing β cells, or highly proliferating malignant cells, which have increased protein synthesis rates, the demand for productive protein folding can be much higher than that for a typical cell. The inward flux of nascent polypeptides into the ER can overwhelm the protein-folding machinery, leading to an imbalance and the accumulation of misfolded protein, which is toxic for the cell. This imbalance is known as “ER stress.” Alongside an increase in protein synthesis, there are a number of factors that give rise to ER stress. These factors include: nutrient deprivation—especially as protein folding is an energy-expending process; deficiencies in post-translational modifications; aberrations in calcium levels and redox homeostasis; inefficiencies in degradation pathways such as ERAD and autophagy; lipid bilayer stress; and low oxygen levels that result in hypoxia.

In order to restore protein folding capacity with protein synthesis requirements, a coordinated transcriptional and translational network termed the unfolded protein response (UPR) is initiated. The UPR monitors protein folding levels within the ER and readjusts folding capacity to match synthesis load, thus ensuring a successful balance for protein homeostasis (Wang and Kaufman, 2016).

A critical step in UPR signaling is the initial detection of ER stress, the process by which unfolded and misfolded proteins are recognized by UPR, leading to activation and downstream signaling. In this review, we discuss the molecular mechanisms that underlie this recognition process, and how this signal is then propagated to the cytosol.

## UPR Signaling

**Figure 1:**
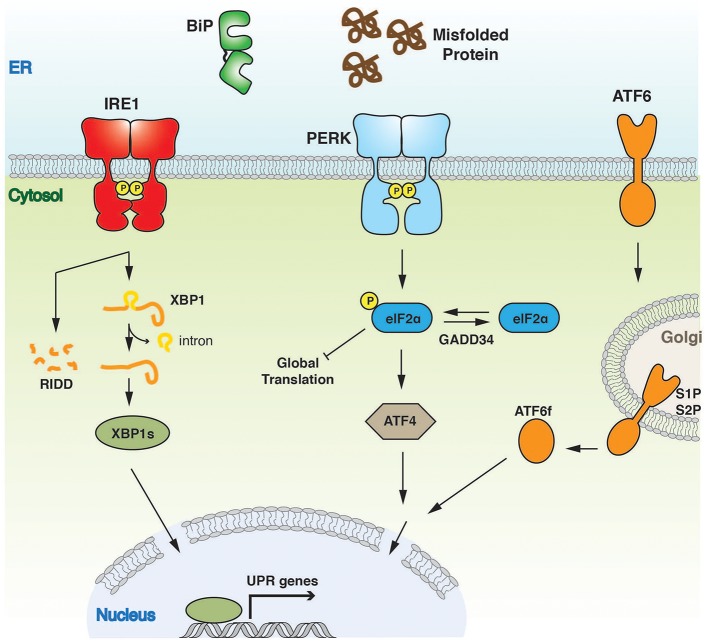
Overview of UPR signaling pathway. The UPR instigates a transcriptional and translational response to ER stress. The three UPR activator proteins, IRE1, PERK, and ATF6 give rise to three separate branches of the response, all of which aim to alleviate the burden of misfolded protein and to ensure successful ER protein homeostasis.

**Figure 2:**
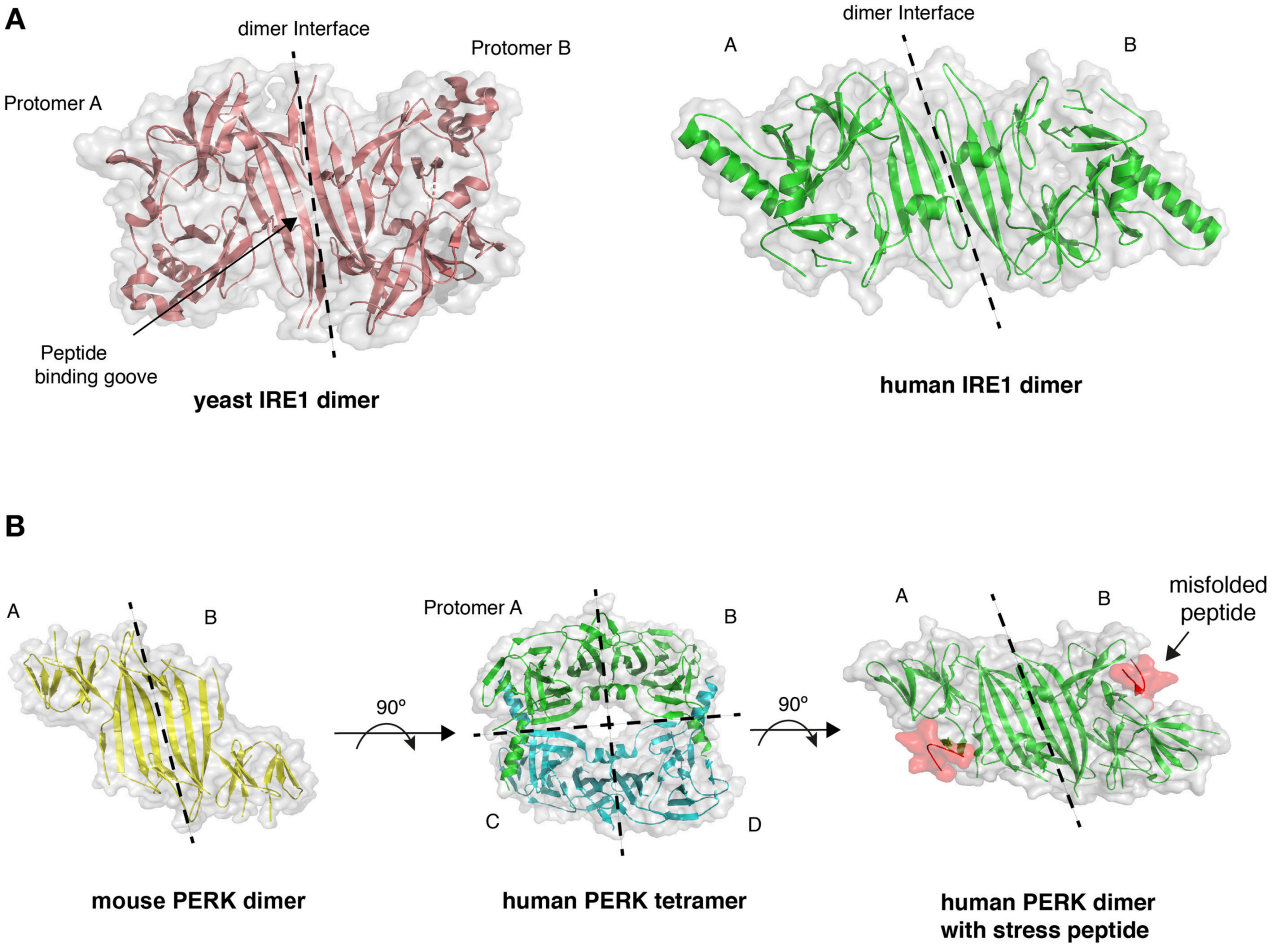
Crystal structures of LD. **(A)** The dimer arrangement of IRE1 LD from both yeast (PDB 2BE1) and human (PDB 2HZ6) proteins, with dimer interface marked by dashed line. **(B)** PERK LD dimer structure shares similar architecture to IRE1 LD. PERK LD has also been visualized in a tetramer arrangement comprising two sets of dimers (PDB 4YZS and 4YZY), and PERK LD bound to peptide (PDB 5V1D).

In metazoans there are three key UPR signal activator proteins: inositol requiring enzyme 1α/β (IRE1) (Cox et al., 1993; Mori et al., 1993), PKR-like ER kinase (PERK) (Harding et al., 1999), and activating transcription factor 6α/β (ATF6) (Haze et al., 1999) (Figure 1). They consist of three domains: an ER luminal domain (LD), a single pass membrane spanning domain, and a cytosolic domain. The domain organization enables the proteins to traverse the ER membrane into the cytosol, with the LD either directly or indirectly involved in sensing misfolded proteins (Walter and Ron, 2011). PERK and IRE1 LD share sequence and structural similarity. Crystal structures of yeast (Credle et al., 2005) and human IRE1 LD (Zhou et al., 2006), along with crystal structures of PERK LD from both mouse and human species (Carrara et al., 2015a) display similar architecture (Figure 2), thus suggesting a similar mechanism of action for both IRE1 and PERK that is conserved from yeast to humans. The cytosolic portion of IRE1 and PERK both possess kinase domains that autophosphorylate in trans (Shamu and Walter, 1996; Tirasophon et al., 1998; Harding et al., 1999; Prischi et al., 2014). For IRE1, this leads to the stimulation of endoribonuclease activity and the splicing of X-box-binding protein 1 (*XBP1)* mRNA to form a potent transcriptional activator, XBP1s (s refers to the spliced form) (Cox and Walter, 1996; Sidrauski and Walter, 1997; Calfon et al., 2002). This results in the upregulation of UPR-targeted genes that not only increase the cells' capacity for protein folding, but also protein degradation and transport pathways, which help to alleviate the burden of misfolded protein within the ER. IRE1 activation can lead to promiscuous endoribonuclease activity, which causes mRNA decay at the ER membrane, thus helping to further reduce the protein load in a process called regulated IRE1 dependent decay (RIDD) (Hollien and Weissman, 2006).

PERK regulates the translation response of the UPR. PERK kinase activation leads to phosphorylation of eukaryotic translation initiation factor-2α (eIF2α), a component of the EIF2 complex, which results in ribosome inhibition and brief attenuation of global cell translation (Harding et al., 1999). Again, this helps in reducing the demands placed on the protein folding machinery. Although PERK activation results in the temporary attenuation of general protein synthesis, paradoxically, certain genes are upregulated, such as activation transcription factor 4 (ATF4) (Vattem and Wek, 2004). The expression of this gene directs an antioxidant response and contributes to a greater ER protein folding capacity.

The third member of UPR signal activators, ATF6, mediates a transcriptional response that promotes protein folding and ER-associated degradation pathways with a similar outcome to IRE1-XBP1 transcriptional activation (Yoshida et al., 2001). However, ATF6 contrasts significantly from both IRE1 and PERK in primary amino acid sequence, domain architecture, and mode of operation. Upon accumulation of misfolded proteins, ATF6 transits to the Golgi apparatus where it is cleaved by site-specific proteases S1P and S2P (Haze et al., 1999; Shen et al., 2002). This releases its cytosolic portion—a bZIP transcription factor—which migrates to the nucleus and mediates activation of UPR targeted genes, such as chaperones.

### Chronic ER Stress and Apoptosis

The primary goal for the UPR is to restore ER protein homeostasis toward ensuring cell survival. However, persistent activation, caused by unmitigated severe ER Stress, leads to a signaling switch that favors apoptosis and a cell death output. Sustained activation of PERK leads to the upregulation of C/EBP-homologous protein (CHOP), a transcription factor implicated in the regulation of apoptosis. This, in turn, leads to the expression of the DNA damage-inducible protein 34 (GADD34), a factor that reverses eIF2α phosphorylation, thereby relieving translational inhibition and enabling the expression of genes, including those involved in ER stressed-induced apoptosis (Novoa et al., 2001).

The IRE1 arm of UPR is geared toward contributing to cell survival, but persistent activation can lead to it interacting with the tumor necrosis factor receptor-associated factor 2 (TRAF2), and inducing an apoptotic output. The interaction with TRAF2 results in the activation of apoptosis signal-regulating kinase (ASK-1) and downstream target c-jun NH2 terminal kinase (JNK) and p38 MAPK. JNK phosphorylation results in the stimulation of pro-apoptotic factors BID and BiM, whilst inhibiting anti-apoptotic factors BCL-2, BCL-XL and MCL-1 (Almanza et al., 2018).

### ER Hsp70 Chaperone: BiP—A Proximal Component of UPR Signaling?

BiP is the sole ER Hsp70 chaperone and one of the most abundant proteins within the ER, making it a major driving force for protein folding. Active BiP levels within the ER are carefully regulated by oligomer formation, post-translational modification such as AMPylation, and UPR induction (Preissler and Ron, 2018). Interestingly, BiP has also been directly implicated in UPR signaling (see below section ER stress sensing by IRE1). It comprises a classical Hsp70 architecture with a nucleotide-binding domain (NBD) and a substrate-binding domain (SBD) that is connected via a linker. BiP operates a typical Hsp70 chaperone substrate mechanism that involves cycling between an open ATP and closed ADP bound state, facilitated by co-chaperones (Kampinga and Craig, 2010; Hartl et al., 2011). Misfolded proteins are recruited to BiP SBD by a certain J-domain containing ERdj co-chaperones, when BiP is present in the open ATP bound state (high K_on_, K_off_). ERdj association stimulates BiP ATPase activity and leads to BiP converting to a closed ADP bound state that traps the misfolded protein substrate (low K_on_ and K_off_). Nucleotide exchange factors (NEF) promote the exchange of ADP to ATP, with BiP reverting to open ATP form that enables the release of bound substrate (Behnke et al., 2015). Thus, BiP is dependent on co-chaperones for its protein folding ability.

## ER Stress Sensing by IRE1

The principal function of the LD is to recognize misfolded proteins within the ER and translate that signal across the membrane to the cytosol. Whether the recognition of misfolded proteins occurs directly by IRE1 LD, or indirectly via the chaperone BiP, is contentious and unclear.

There are two established models, the “direct association” and BiP “competition,” that seek to explain how misfolded proteins induce UPR. More recently, an alternative BiP “allosteric” model has been proposed.

### Direct Association Model

**Figure 3:**
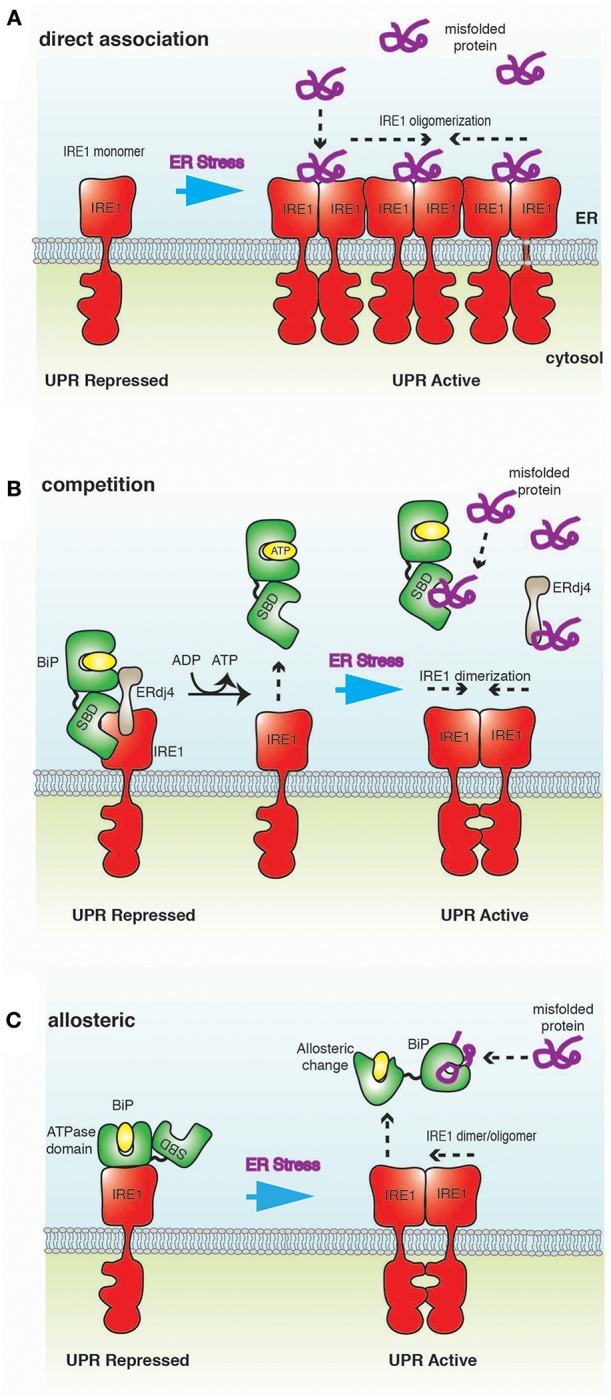
A schematic representation of ER stress-sensing mechanisms. **(A)** Direct association model posits that misfolded proteins bind directly to IRE1 LD, resulting in oligomerization of IRE1 and activation of UPR. **(B)** In the competition model, IRE1 LD binds to BiP SBD in a chaperone-substrate type interaction. This is the same site that misfolded proteins bind to BiP, leading to a competition for this binding site. BiP interaction to IRE1 is mediated by ERdj4, which ultimately inhibits UPR signaling by facilitating the formation of IRE1 LD monomer. Thus, BiP acts as a repressor of UPR signaling, but is not a direct sensor of ER stress. **(C)** In the allosteric model, the binding of misfolded proteins and IRE1 LD to BiP occur on different domains; thus, obviating the requirement for competition. Misfolded protein binding induces a conformational change that releases BiP from IRE1, implicating BiP as a direct sensor of ER stress.

The direct association model postulates that misfolded proteins bind directly to the LD of IRE1 to activate UPR signaling (Credle et al., 2005; Kimata et al., 2007; Gardner and Walter, 2011; Promlek et al., 2011; Karagöz et al., 2017) (Figure 3A). The association of misfolded protein mediates conformational changes that result in the oligomerization of IRE1 LD and subsequent activation of UPR signaling (Credle et al., 2005; Karagöz et al., 2017; Karagoz et al., 2019). In this model, BiP is not involved in detecting ER stress, but plays a peripheral role by binding and sequestering inactive monomeric IRE1 (Pincus et al., 2010).

The direct association model is based on crystal structures of LD that suggest the formation of a peptide binding groove upon dimerization that resembles a major histocompatibility complex (MHC)-like fold (Credle et al., 2005). Mutation of residues within this groove impaired IRE1 signaling in yeast (Credle et al., 2005; Gardner and Walter, 2011). A peptide tiling array analysis identified peptides that interacted with yeast IRE1 LD *in vitro* that displayed a distinct amino acid composition similar to exposed polypeptide stretches found within the hydrophobic core of a protein (Gardner and Walter, 2011; Karagoz et al., 2019). The interaction with peptides also caused an increase in the LD oligomer species (Gardner and Walter, 2011). More recently, a structural and biochemical analysis indicated that human IRE1 LD was able to bind to both peptides and unfolded proteins *in vitro*, thus displaying similarities with yeast IRE1 LD (Karagöz et al., 2017). Also, nuclear magnetic resonance (NMR) experiments suggest conformational changes upon the binding of peptide to the MHC-like groove, with these conformational changes thought to facilitate IRE1 oligomerization and UPR activation (Karagöz et al., 2017). A recent crystal structure of PERK LD bound to misfolded peptide suggests that PERK can also bind to misfolded proteins (Figure 2B) (Wang et al., 2018).

BiP role in the direct association model is to fine-tune the activity of IRE1 sensor. A study that utilized a series of IRE1 deletion mutations suggested the binding site for BiP was proximal to the membrane (Kimata, 2004). Mutation of this site did not cause unrestrained UPR activation but displayed reaction kinetics consistent with BiP acting as a buffer of IRE1 activity in yeast (Kimata, 2004; Pincus et al., 2010; Karagoz et al., 2019).

However, although peptides bind to IRE1 LD, it is not clear whether the association was occurring at a single site or at multiple sites with less specificity (Preissler and Ron, 2018). Interestingly, the only crystal structure of LD bound to a peptide indicated that the peptide did not associate to the MHC-like groove but to a hydrophobic pocket that is required for PERK tetramer formation. This pocket is unlikely to have a stress-sensing role and it is surprising that the peptide did not bind to the MHC-like groove in this structure (Figure 2B) (Wang et al., 2018). Moreover, from an evolutionary perspective, it is difficult to rationalize why IRE1 would not evolve to utilize BiP as an ER stress detector (and not only as a buffer of IRE1 activity), especially since it interacts with BiP, whose primary function is to bind to misfolded proteins.

### Competition Model

In this model, BiP binds IRE1 LD as a chaperone-substrate type interaction via its SBD to form a repressive complex (Bertolotti et al., 2000; Okamura et al., 2000; Ma et al., 2002; Kimata et al., 2003; Oikawa et al., 2009; Amin-Wetzel et al., 2017; Preissler and Ron, 2018) (Figure 3B). This interaction is mediated by ERdj4 and occurs on the same site that misfolded proteins bind to BiP. The formation of this complex stimulates BiP ATPase activity, resulting in ERdj4 dissociation and causing IRE1 LD to form monomers, leading to inhibition of UPR signaling (Amin-Wetzel et al., 2017). NEF facilitate the exchange of ADP to ATP, causing BiP to dissociate from IRE1 LD. Misfolded proteins now compete with IRE1 LD for binding to both free BiP and ERdj4. In high ER stress, BiP and ERdj4 become occupied with engaging misfolded proteins, thus impeding BiP association with IRE1 LD. This enables IRE1 to form dimers, which in turn activates UPR signaling (Amin-Wetzel et al., 2017). In this model, BiP acts as a repressor of UPR signaling by preventing dimerization of IRE1, but not as a direct ER stress sensor.

The central tenet of the competition model is that the binding between IRE1 and BiP is a chaperone-substrate type interaction that occurs via BiP SBD, the same site that misfolded proteins bind to BiP, resulting in a competition for this site (Preissler and Ron, 2018). This is analogous to competitive repression of the transcription factor, heat shock factor 1 (Hsf1), activity by Hsp70 in the cytosol (Abravaya et al., 1992). In the competition model, the actions of BiP are firmly based on the principles of nucleotide-dependent regulation of Hsp70 when it interacts with chaperone-substrate (Hartl et al., 2011; Mayer, 2013). An important facet of the mechanism is that ATP causes BiP dissociation from IRE1, and not misfolded proteins. This is in keeping with Hsp70-ATP being the substrate loading and unloading state of the chaperone. Additionally, NEF facilitate the exchange of ADP to ATP, similar to how nucleotide exchange is achieved with the Hsp70 chaperone system. Moreover, ERdj4 functions as a recruitment factor that mediates interaction between BiP and IRE1, akin to how certain ERdj proteins recruit misfolded proteins to BiP (Behnke et al., 2015). Thus, in this model the interaction between IRE1 and BiP is governed by the principles of how Hsp70 interacts with a chaperone-substrate.

However, if the binding between IRE1 and BiP were not a chaperone-substrate type interaction and were to occur at another site on BiP i.e., the NBD, then the competition model would not hold true. This is due to the fact that it obviates the requirement for competitive binding between IRE1 and misfolded protein for BiP SBD and a nucleotide-dependent mechanism that underlie how Hsp70 interacts with chaperone-substrate (see below—Allosteric model). Also, contrary to this model, a number of studies have observed binding between BiP and IRE1 independent of ERdj4 *in vitro* (Carrara et al., 2015b; Kopp et al., 2018; Sepulveda et al., 2018). Furthermore, the current model suggests that there will be no difference in binding between folded IRE1 LD (LD has a high degree of secondary structure) and misfolded IRE1 LD to BiP.

### Allosteric Model

The allosteric model indicates an interaction between BiP NBD and IRE1 LD (Todd-Corlett et al., 2007; Carrara et al., 2015b; Kopp et al., 2018) (Figure 3C). This interaction is independent of nucleotides (Carrara et al., 2015b; Kopp et al., 2018) and is distinct from the chaperone-substrate type interaction that occurs via BiP SBD (Amin-Wetzel et al., 2017). Misfolded proteins bind exclusively to the canonical BiP SBD, which leads to dissociation of BiP NBD from IRE1 LD via a conformational change to trigger UPR signaling (Carrara et al., 2015b; Kopp et al., 2018). As misfolded proteins and IRE1 LD bind to different domains of BiP, there is no requirement for this process to be competitive. In this model, BiP acts as a direct sensor of ER stress (Carrara et al., 2015b; Kopp et al., 2018).

Criticism of this model is centered on the observation that ATP does not cause the dissociation of BiP from IRE1, leading to the suggestion that the model does not obey the principles of nucleotide-dependent Hsp70 regulation (Preissler and Ron, 2018). This would be true if the interaction between IRE1 and BiP were a chaperone-substrate type interaction; however, in this model binding occurs via the BiP NBD. Hsp70 chaperones are primarily concerned with the protein folding processes, but they can specialize in certain roles within this remit, for example, in protein translocation (Craig, 2018). An analogy can be made with mitochondrial Hsp70 (mtHsp70) action during the translocation of polypeptide into the inner mitochondrial matrix. mtHsp70 associates with Tim44, a component of the translocon machinery, whilst awaiting the import of nascent polypeptide (Craig, 2018). The interaction with Tim44 primarily occurs via mtHsp70 NBD with contributions from the mtHsp70 SBD (but not as a chaperone-substrate interaction as it is still able to bind to misfolded peptide whilst bound to Tim44) (Krimmer et al., 2000; D'Silva et al., 2004). More significantly, the interaction is independent of nucleotides (Liu et al., 2003). This is based on the observation that the addition of ATP and ADP failed to cause dissociation of the mtHsp70-Tim44 complex *in vitro*. It is only the addition of peptides binding to mtHsp70 SBD that caused mtHsp70 to release from Tim44 (Liu et al., 2003). Similarly, the interaction of IRE1 LD to BiP NBD is independent of nucleotides *in vitro* (Carrara et al., 2015b; Kopp et al., 2018). The binding affinity between IRE1 LD and BiP in the absence or presence of nucleotides (ATP, ADP and AMP-PNP) were closely comparable (K_d_ 1–2 μM). It is only with the addition of misfolded protein (C_H_1), binding to BiP SBD, that caused BiP to dissociate from IRE1 via BiP NBD (Carrara et al., 2015b; Kopp et al., 2018). The observation that both mtHsp70 and ER Hsp70 interact with membrane-associated proteins via their NBD, and that the interaction is not influenced by nucleotides, with only misfolded protein/peptide binding to SBD causing dissociation, suggests mechanistic similarities between these two chaperones. Thus, the role of BiP in the allosteric model fits with known principles of Hsp70 mechanistic action, particularly when operating in specialized roles that interact with partner proteins in a non-chaperone-substrate type fashion.

However, there are a number of points yet to be addressed, such as whether nucleotides influence other aspects of the allosteric model, in particular, the ability of BiP to engage misfolded substrate whilst bound to IRE1 LD. Again, clues can be gleaned from the mtHsp70 system. The interaction between mtHsp70 and Tim44 is unaffected by nucleotides *in vitro* (Liu et al., 2003), but coimmunoprecipitation of mtHsp70-Tim44 complex from mitochondrial lysates were greatly sensitive to ATP and dissociated the complex (Krimmer et al., 2000; Liu et al., 2003). This is because there were polypeptides with exposed hydrophobic amino acids within the lysate that were able to associate with mtHsp70-Tim44 to cause dissociation of complex, and ATP enhanced the engagement of misfolded polypeptide substrates via SBD, without directly impacting the mtHsp70(NBD)-Tim44 interaction itself. A similar scenario likely occurs with BiP-IRE1 LD. This interaction is independent of nucleotides *in vitro* (Carrara et al., 2015b; Kopp et al., 2018), but seems to be sensitive to ATP in cell lysate (Bertolotti et al., 2000). So, does ATP sensitize the BiP-IRE1 LD complex to engage misfolded proteins via BiP SBD leading to enhanced dissociation, without directly impacting BiP(NBD)-IRE1 LD interaction? Another interesting point to investigate is: how does BiP operate as a molecular chaperone and as an ER stress sensor? The allosteric model suggests that there will be differences in the way BiP interacts with folded IRE1 LD (via BiP NBD) and misfolded IRE1 LD (via SBD).

### Oligomerization

After the detection of misfolded proteins, the signal is propagated across the ER membrane via a change in the oligomeric state of IRE1 and PERK, engendering cytosolic domain activation. There are numerous reports regarding the oligomeric state of LD and its transition upon activation induced by ER stress, including monomer to dimer transitions (Shamu and Walter, 1996; Welihinda and Kaufman, 1996; Bertolotti et al., 2000; Liu et al., 2000; Okamura et al., 2000; Oikawa et al., 2005; Zhou et al., 2006; Lee et al., 2008; Carrara et al., 2015b; Amin-Wetzel et al., 2017), tetramer formation (Carrara et al., 2015a), and higher oligomers (Credle et al., 2005; Kimata et al., 2007; Aragón et al., 2008; Korennykh et al., 2008; Gardner and Walter, 2011; Sundaram et al., 2018). Thus, it is likely that the LD is the primary determinant of IRE1 oligomeric status in response to ER stress, with contributions from both the transmembrane and cytosolic regions.

Overall, the mechanism by which IRE1 LD senses ER stress and induces UPR signaling is still not clearly understood. There are three models that provide contrasting mechanisms to explain how ER stress is detected. However, some aspects of these models are not mutually exclusive and could possibly operate synergistically (Kimata et al., 2007). Further studies are required to differentiate or reconcile between the contrasting models and to understand how this affects IRE1 oligomerization leading to cytosolic signal propagation.

### Modulators of IRE1 Stress Signaling in the ER Lumen

Emerging evidence suggests that there may be factors that can bind to the IRE1 LD and influence its ability to detect or respond to ER stress. An ER resident PDI (PDIA6) has been suggested to attenuate UPR signaling by binding to IRE1 LD and reducing a disulphide bond. The oxidized form of the disulphide bond was associated with oligomer formation and UPR activation. The reduction of the bond facilitates the transition from oligomeric to monomeric IRE1, thereby preventing downstream IRE1 kinase phosphorylation and UPR signaling. In a similar fashion, PDIA6 also binds to PERK LD and attenuates its signaling, but does not interact with ATF6 and thus has no direct effect on this branch of UPR signaling (Eletto et al., 2014).

More recently, a study has suggested an interaction between IRE1 LD and the ER chaperone, Hsp47 (Sepulveda et al., 2018). Hsp47 belongs to the serine-protease (serpin) inhibitor family. It functions by binding to collagen and trafficking it from the ER to the Golgi in a pH-dependent fashion. Surprisingly, Hsp47 binds to IRE1 LD with high affinity and displaces BiP. This releases UPR repression by allowing the formation of IRE1 dimers to activate signaling.

The lipid composition of the ER membrane may also modulate UPR signaling. IRE1 contains an amphipathic transmembrane helix that is suggested to respond to different membrane lipid compositions by eliciting oligomerization in certain conditions. ATF6 has also been shown to be activated by lipids. In both cases, lipid-based activation occurs independently of proteotoxic stress mechanisms (Volmer et al., 2013; Halbleib et al., 2017; Tam et al., 2018).

Proteins that bind directly to IRE1 LD, along with membrane lipids, may modulate UPR signaling by ER stress-independent mechanisms. This may provide an extra level of regulation in which the UPR signal could be attenuated or strengthened. However, their exact integration with current ER stress-sensing mechanisms that regulate UPR signaling and ER homeostasis is yet to be determined.

## IRE1 Cytosolic Stress Signaling

Upon ER stress, the UPR signal is propagated to the cytosolic portion via a change in its oligomeric status, stimulating IRE1 kinase and subsequently RNase activity. Both IRE1 and PERK cytosolic portions contain kinase domains that autophosphorylate in trans, suggesting that dimerization/oligomerization is required for activation and signaling.

### IRE1 Autophosphorylation Crystal Structure

**Figure 4:**
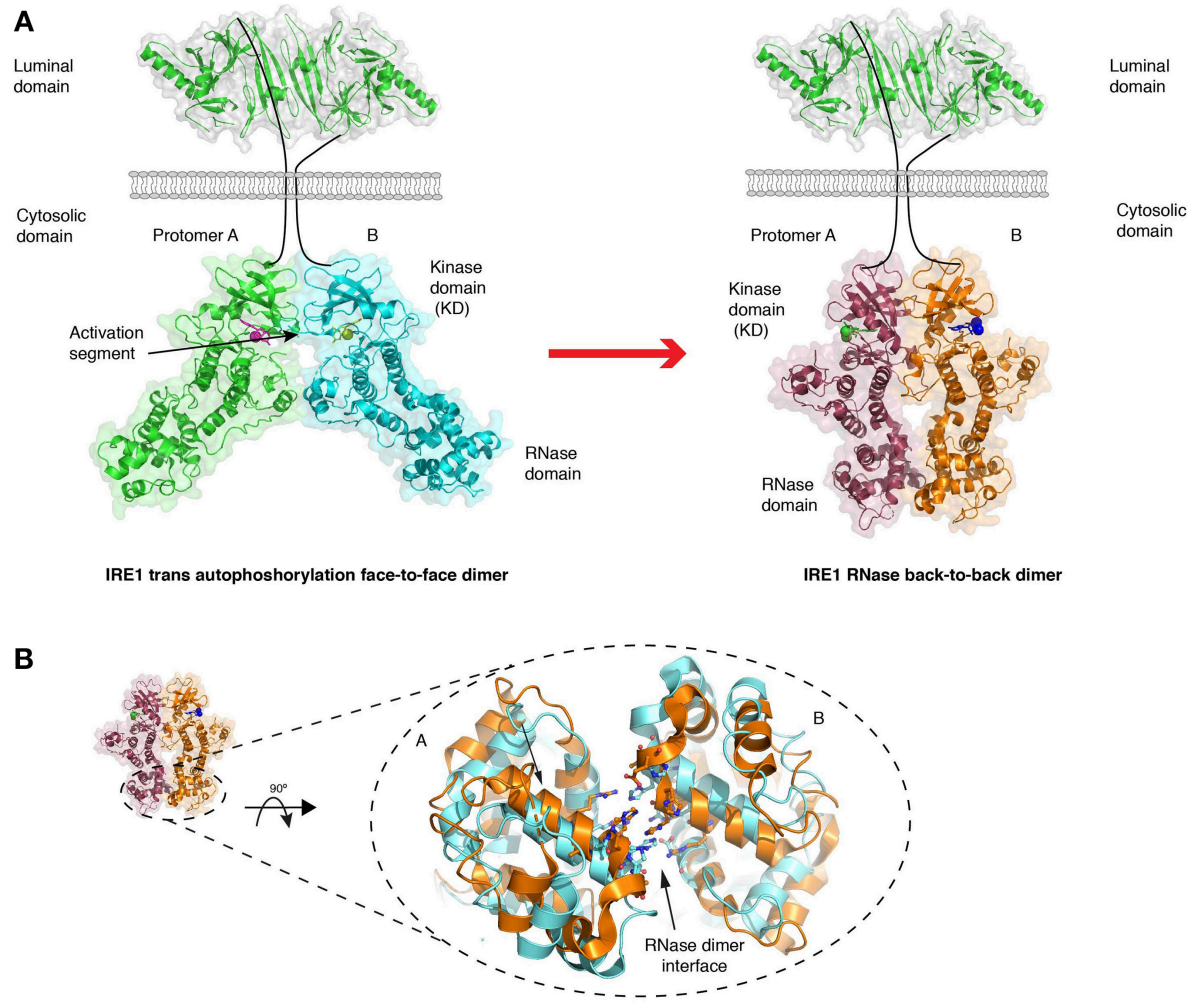
Crystal structures of IRE1 cytosolic domain. **(A)** Schematic depicting the IRE1 cytosolic portion in a face-to-face dimer (PDB 3P23) that enables trans autophosphorylation, and in a back-to-back arrangement (PDB 2RIO), which is suggested to be the RNase active state. The red arrow represents the transition between these two states. **(B)** A comparison of crystal structures of IRE1 RNase domain when bound to a kinase inhibitor that prevents both kinase and RNase activation (gold, PDB 4YZ9) and when bound to a kinase inhibitor that activates RNase domain (cyan, PDB 4YZC). The small movements within the domain are suggested to enhance splicing activity.

The crystal structure of the human cytosolic portion of IRE1 displays a dimer arrangement with each monomer orientated such that their kinase active sites face toward each other (Ali et al., 2011) (Figure 4A). In this face-to-face orientation, the kinase activation loop—the loop that is phosphorylated—points toward the active site of the opposing monomer in a manner that would allow autophosphorylation in trans to occur. This is reminiscent of similar kinases that have been structurally characterized to undergo dimerization dependent activation, including Chk2 and Lck (Oliver et al., 2007; Pike et al., 2008). The face-to-face orientation provides a rationale to how reciprocal autophosphorylation upon the activation loop may occur. In this particular crystal structure, IRE1 was de-phosphorylated, but a similar face-to-face arrangement has been described for phosphorylated murine IRE1 crystal structure (Sanches et al., 2014).

### IRE1 RNase Active Crystal Structures

The cytosolic portion of IRE1 has also been crystallized in an alternative dimeric arrangement (Lee et al., 2008; Concha et al., 2015; Joshi et al., 2015) (Figure 4A), in which their kinase active sites face away from each other, enabling a more substantial contact between the RNase domains of the monomers (Lee et al., 2008). This back-to-back arrangement may represent the RNase activated form. Within these structures, there were differences in the way the RNase domain aligned with each other depending on whether the kinase domain was in an active or inactive/inhibited conformation (Concha et al., 2015; Joshi et al., 2015). This resulted in small differences in the hydrogen bonding network between RNase monomers, with more substantial interactions favoring higher splicing activity, thus suggesting that RNase dimer interface movements could provide contrasting splicing outputs (Concha et al., 2015; Joshi et al., 2015) (Figure 4B). Aside from the dimer state of the cytosolic domain, there is a crystal structure of yeast IRE1 cytosolic domain forming a large helical arrangement utilizing the dimer as a building block (Korennykh et al., 2008), which may represent oligomerization events upon ER stress.

So far, crystal structures provide the basis for mechanistic interpretation, but there is still the need for greater clarity. IRE1 cytosolic portion predominantly forms dimers without the influence of the LD. The dimer has been visualized in two separate orientations that seem to be distinct from each other (Lee et al., 2008; Ali et al., 2011). It is plausible these arrangements are interconvertible, with the initial state being the autophosphoryl competent face-to-face orientation. This is in keeping with the requirement for phosphorylation to occur at the activation loop to stimulate RNase activity (Prischi et al., 2014), which then transits to the back-to-back arrangement, and consequently larger oligomeric structures (Joshi et al., 2015). Although how this would work remains to be resolved, particularly since the cytosolic domain rearrangements would depend upon the LD, and thus far the LD dimer structures seem to suggest that it is present in only one stable form. Additionally, why the RNase domains are required to interact, considering that the core catalytic residues are present in each monomer, remains to be elucidated.

### Inhibitors of IRE1 Enzymatic Activity

IRE1 ability to influence cellular fate has made it a target for pharmacological intervention in disease. Chemical compounds that modulate IRE1 activity specifically target its kinase or endoribonuclease enzymatic function in order to influence the levels of spliced XBP1. So far, small molecules that target the RNase activity are aldehyde derivatives that covalently modify the active site leading to inhibition (Papandreou et al., 2011; Volkmann et al., 2011; Cross et al., 2012; Mimura et al., 2012; Sanches et al., 2014). These compounds directly interact with the lysine 907, a key RNase catalytic residue (Tirasophon et al., 2000), forming a stable imine that prevents XBP1 splicing.

Small molecule inhibitors that target IRE1 kinase activity work by competitively binding to the kinase active site and displacing ATP, thereby preventing the kinase trans autophosphorylation reaction. However, these ATP competitive inhibitors have differing effects on the RNase activity, with some compounds increasing RNase splicing (Papa et al., 2003; Korennykh et al., 2008; Concha et al., 2015; Feldman et al., 2016), and others inhibiting RNase activity (Wang et al., 2012; Concha et al., 2015). A subset of compounds that inhibit activity were based on imidazopyrazine scaffold and were termed kinase inhibiting RNase attenuators (KIRA) (Wang et al., 2012). Another study reported inhibition by a compound with a spirodecane core (Concha et al., 2015).

Mechanistically, ATP competitive inhibitors impact kinase activity by displacing key secondary structural elements (αC helix and DFG motif) within the active sites that are required for productive phosphorylation to occur. This results in the kinase domain adopting an inactive conformation, with corresponding realignment of the RNase domain and inhibition of RNase splicing (Concha et al., 2015). The engagement of KIRA compounds to the kinase active site prevents the formation of dimers, keeping IRE1 in a monomeric state (Wang et al., 2012). Surprisingly, KIRA compounds seem to be able to dictate the IRE1 oligomerization status. This suggests that IRE1 C-terminal stimulus may regulate LD multimer formation, in contrast to ER stress-induced LD oligomerization, and may represent a novel and unusual method for communicating from cytosol to ER.

## IRE1 Cytosolic Domain Interacting Proteins

There are a growing number of studies that have identified proteins that interact with IRE1 cytosolic portion to influence UPR signaling output. There are three categories that the IRE1 interacting proteins fall into: proteins that inhibit IRE1 signaling; proteins that activate IRE1 signaling; and proteins that bind to IRE1 as a scaffold and recruit other proteins.

### Inhibitory Interactions

Interacting proteins that exert an inhibitory effect include the apoptosis- and tumor-linked factor: Fortilin. Binding between Fortilin and IRE1 occurs only when IRE1 is phosphorylated, and its interaction inhibits both kinase and RNase activity, possibly by blocking IRE1 dimer formation and autophosphorylation (Pinkaew et al., 2017). This action attempts to prevent IRE1-induced apoptosis signaling, by reducing phosphorylated IRE1 levels. In a similar manner, the apoptosis regulator Bax inhibitor 1 (BI-1) has been suggested to interact with IRE1 and again inhibits apoptosis signaling. In contrast to Fortilin, this attenuation is achieved by obstructing TRAF2 binding (Castillo et al., 2011). The underlying inhibitory mechanism here is to prevent dimer formation or to displace other interacting proteins.

### Stimulatory Interactions

Proteins that have been suggested to stimulate IRE1 activity include Abelson tyrosine protein kinase 1 (ABL1 or c-abl), a tyrosine kinase implicated in a diverse range of cell signaling processes. Under stress conditions, it engages the cytosolic portion of IRE1 to induce oligomerization, leading to hyperactivation of the RNase function (Morita et al., 2017). Similarly, Non-muscle myosin-IIb (NMIIB) and Filamin A, are two proteins that are involved in actin cytoskeleton remodeling. NMIIB interaction is dependent on ER stress and leads to IRE1 oligomerization (He et al., 2012). Filamin A interaction is independent of ER stress and IRE1 stress signaling; its binding to monomeric IRE1, at a distal C-terminal region, possibly leads to dimer formation and recruitment of PKC, which enables Filamin A phosphorylation (Urra et al., 2018). This acts to increase actin cytoskeletal remodeling. Thus, IRE1 stimulatory proteins are suggested to shift the monomer state to a higher oligomeric assembly.

### Scaffold Interactions

Scaffold proteins that engage IRE1 include TRAF2 (Urano et al., 2000). Its binding facilitates the recruitment of JNK to IRE1 and influences an apoptotic outcome by signaling via the ASK1 pathway and caspase cascade (Urano et al., 2000; Castillo et al., 2011). TRAF2 recognizes and specifically engages the phosphorylated IRE1, although it is not known how this occurs. Moreover, what influences scaffold proteins have upon the oligomeric status of IRE1 is not known. Other IRE1 scaffold proteins include; Nck, a cell signaling adaptor protein, which recruits Nuclear Factor κB (NF-κB) (Nguyen et al., 2004); and CHIP, an E3 ligase that ubiquitylates IRE1. This modification may enhance TRAF2/JNK signaling, because in CHIP knockdown cells, IRE1 phosphorylation and IRE1-TRAF2 interactions were nearly abolished (Zhu et al., 2014). Interestingly, a recent study has suggested an interaction between IRE1 and Sec61 translocon. The formation of the complex provides a platform for the recruitment of XBP1 mRNA, enabling more efficient splicing by IRE1 at the ER membrane (Plumb et al., 2015). The molecular details of this interaction are yet to be determined and its elucidation could possibly provide further molecular clues into IRE1 splicing activity.

Proteins that interact with the IRE1 cytosolic region may provide an auxiliary way of modulating signaling output and cell fate. Also, scaffolding proteins provide a way to link IRE1 and UPR signaling to other signaling networks and processes. However, the details of such interactions are yet to be determined and highlight the need for a better understanding of the molecular mechanism.

## Conclusions

Great progress has been made toward understanding the mechanism of IRE1 stress signaling since its role was first described as mediating a rectifying signal that restores ER homeostasis. Crystal structures have formed the basis of our molecular understanding. The general approach has been to dissect IRE1 into its component parts—the LD and the cytosolic domain—based on the cellular compartment to which they originate from, toward understanding the roles that IRE1 plays in ER stress recognition and UPR signal propagation. The mechanism by which IRE1 detects ER stress is still not clearly understood with three alternative models put forth, highlighting the need for further studies that either provide support or offer reconciliation between models. The cytosolic domain structures have helped to inform and guide drug development programs that aim to target IRE1 kinase and RNase activity, and have provided substantial insights into the mechanism. However, additional experiments are required to provide more molecular detail into IRE1 enzymatic activity. Future structural studies would benefit from understanding how the two domains, residing in two separate cellular compartments, communicate with each other in the absence and presence of ER stress. This communication may be coupled to transitions in oligomeric status of IRE1, emphasizing the importance of understanding this mechanistic step. Also, it would be very interesting to learn the molecular basis of how modulators that bind both the IRE1 LD and cytosolic domain influence output. Overall, although significant progress has been made toward the understanding of IRE1 stress signaling, there still remain many unresolved questions that require further experimentation. Such research may yet provide significant and novel mechanistic insights and discoveries into the IRE1 function.

## Author Contributions

CA, MK, NL, PN, and MA read and reviewed final version of the work, and participated in bibliographical research and design. CA assisted in writing and figure preparation. MA wrote paper and did the figures.

### Conflict of Interest Statement

The authors declare that the research was conducted in the absence of any commercial or financial relationships that could be construed as a potential conflict of interest.
